# Wrist extensor fatigue and game-genre-specific kinematic changes in esports athletes: a quasi-experimental study

**DOI:** 10.1186/s13102-025-01305-0

**Published:** 2025-09-01

**Authors:** Chuck Tholl, Lasse Hansen, Ingo Froböse

**Affiliations:** 1 Institute of Movement Therapy and Movement-oriented Prevention and Rehabilitation, German Sport University Cologne, Cologne, Germany; 2 Intitute of Biomechanics and Orthopaedics, German Sport University Cologne, Cologne, Germany

**Keywords:** Video games, Overuse syndrome, Biomechanics, Motion capture, Screen-based activity, Physical demands

## Abstract

**Background:**

Muscular fatigue critically affects health, performance, and safety in daily activities and sports. Esports or competitive gaming involves prolonged sitting and repetitive upper extremity movements, increasing the risk of muscular fatigue. Sustained activity may contribute to long-term musculoskeletal disorders (MSD). Despite this risk, biomechanical analyses in esports remain limited. This study examines muscular fatigue and wrist kinematics in esports athletes across different video game genres.

**Methods:**

Thirty-two healthy male esports athletes (23.8 ± 3.4 years) participated in two 90–120-minute competitive video gaming sessions, separated by a 10-minute passive sitting break. Surface electromyography (EMG) of the upper trapezius and wrist extensors, as well as wrist kinematics, were recorded. The median frequency (MDF) and root mean square (RMS) were used to quantify muscular fatigue. Statistical analyses included mixed ANOVA, one-way repeated measures ANOVA, and robust ANOVA with Bonferroni correction.

**Results:**

Repeated measures ANOVA indicated significant decreases in the MDF and RMS of the wrist extensors over time (*p* < 0.001). For the upper trapezius, only the right-side MDF showed a significant decrease over time; however, post-hoc analysis did not confirm this effect. Mixed ANOVA revealed no interaction between time and video game genre on kinematic data. First-person shooter players exhibited significantly greater cumulative distances (*p* = 0.006) and velocity zero-crossings (*p* = 0.043) than multiplayer online battle arena players in robust ANOVA.

**Conclusions:**

The findings indicate a progressive increase in wrist extensor fatigue over time, whereas wrist kinematics vary by video game genre but remain unaffected by time. The lack of neuromuscular recovery post-break suggests the potential for cumulative muscular fatigue. These repetitive loads could increase the risk of MSD. Therefore, implementing preventive training strategies and regular active breaks may help mitigate these effects in esports athletes.

**Supplementary Information:**

The online version contains supplementary material available at 10.1186/s13102-025-01305-0.

## Background

Competitive video gaming, known as electronic sports (esports), attract millions of spectators, thousands of esports athletes and is an essential element of today’s youth culture [1]. In contrast to recreational video game players, esports athletes train specifically to reach the highest performance levels and to compete in tournaments against other human players under specific regulations [2]. In particular, esports performance requires psychological-cognitive and communicative abilities as well as mechanical skills [3]. To reach this high stage of performance esports athletes exercise between 4 and 10 h/day depending on their skill level and game genre [4, 5]. As esports and video gaming are predominantly sedentary activities [6], esports athletes are faced with increased sitting times which are associated with several negative health outcomes [7, 8].

Requirements and musculoskeletal loading of esports athletes are comparable to those of other sedentary populations with high cognitive demands, such as computer workers, pianists or air traffic controllers [9, 10]. These populations have a higher risk for work- or practice-related musculoskeletal disorders [11–13]. Those occupational groups perform activities which require repetitive movements of the upper limbs and increased actions per minute (APM) to perform at high levels [10]. In esports, APM refers to the number of mouse clicks and keystrokes used to control the virtual environment. Depending on the game genre, esports athletes can reach over 500 APM [14]. Such repetitive movements pose a risk for muscular fatigue if they are executed continuously without breaks [15, 16]. In the short term this may lead to declines in performance [17, 18] and to an increase in perceived exertion [19]. Over the long term, muscular fatigue may contribute to adverse health outcomes, particularly overuse injuries or musculoskeletal disorders (MSDs) [20, 21]. A systematic review demonstrated that video gaming for three or more hours a day was associated with higher rates of reported MSDs [6]. However, the majority of the included studies were cross-sectional surveys, which limit the causal inference. MSDs result from a combination of factors, with biomechanical stress playing a particularly important role alongside psychosocial, socioeconomic, and environmental risks [12]. These include postural problems, high force exertions, highly repetitive tasks, ergonomic aspects and muscular fatigue [12, 21, 22]. In particular, highly repetitive movements and muscular fatigue have been observed in esports, but have not been rigorously studied [23].

Muscular fatigue is defined as a reduction in maximal force or power production in response to contractile activity and can be separated into central and peripheral components [20, 24]. Whereas central fatigue influences voluntary activation of muscle from within the central nervous system, peripheral fatigue is related to the neuromuscular junction and impair the contractile function of muscle [18]. The impact of video gaming on muscular fatigue has been investigated in only a limited number of studies [25–27]. Two studies demonstrated an increase in muscular fatigue after 20–30 min of smartphone gaming in the back [26] and in thumb muscles [27] of non-gamers. Divergent results were demonstrated after 1 h of mouse clicking task with gamers and non-gamers [25]. The study showed an increase in electrical activity but no alterations in frequency parameters on average, which led the authors to conclude that muscular fatigue was not observed. Overall, monotonous computer work could lead to increased muscular fatigue, if performed continuously [15, 28]. It can be reasonably deduced that the esports environment has the potential to induce muscular fatigue.

One potential contributing factor to muscular fatigue in esports is the kinematic structure of the activity itself. Studies observed faster hand acceleration in professionals compared to amateurs [29, 30]. Additionally, professionals show more accurate hand and elbow movements in the mouse arm [29]. Differences in kinematic patterns can also be seen across various game genres. Specifically, first-person shooter (FPS) and multiplayer online battle arena (MOBA) players showed significantly higher hand accelerations, repetitive motions and larger cumulative travel distances than adventure players [31]. Consequently, such movement patterns could lead to muscular fatigue. However, the existing studies do not fully reflect the real-world conditions in which esports athletes compete. Either only specific aspects of gameplay are tested, or the time periods studied are too short.

Therefore, the main objective of the current study is to explore the effects of competitive video gaming on muscular fatigue and wrist kinematics. The two primary hypotheses are: (1) muscular fatigue increases over time; and (2) wrist kinematics differ between video game genres and across time. The sub-hypothesis is that (3) mouse sensitivity acts as a co-factor influencing kinematic outcomes. The results may offer a better understanding of contributing factors for physical strains in esports. Moreover, these findings may contribute to the enhancement of holistic training practices while also supporting the development and application of preventive and rehabilitative strategies.

## Materials and methods

### Study design

**Fig. 1:**
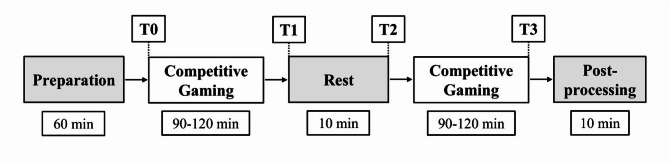
Study design. T0 = before first competitive video gaming sessions; T1 = after first competitive video gaming sessions and before rest; T2 = after rest and before second competitive video gaming sessions; T3 = after second competitive video gaming sessions

This quasi-experimental study followed a repeated-measures, within- and between-group, non-randomized design to minimize individual variability [19]. The study was implemented at the Institute of Movement Therapy and Movement-oriented Prevention and Rehabilitation at the German Sport University Cologne. Between June and December 2023, esports athletes were recruited for a five-to six-hour investigation. They participated in two competitive video gaming sessions of 90–120 min, separated by a 10-minute passive sitting break (Fig. 1). Participants played only their primary game from one of two genres (FPS or MOBA) during both sessions, using their personal gaming accounts. Genre or game switching was not permitted. The protocol followed the ethical principles of the Declaration of Helsinki and was approved by the ethics committee of the German Sport University Cologne (reference: 093/2023).

### Participants

The a priori power analysis estimated a minimum required sample size of *N* = 24 for mixed and repeated measures one-way ANOVA [32], considering a mean effect size (f) of 0.25, a significance level (α) of < 0.05, and a power (1-β) of 0.8. To account for potential participant dropout, we implemented an over-recruitment strategy of approximately 30%. This approach was intended to preserve statistical power and ensure complete data collection despite expected attrition [33]. A total of 32 healthy male esports athletes from Germany, aged between 18 and 35 years, participated in this study. Esports athletes were defined as those ranked in the top 20% of their respective video game’s ranking system. Due to the absence of a standardized definition and classification of esports athletes [34], participants were categorized based on their competitive ranking. Specifically, those in the top 1% were classified as professionals, while all others were considered amateurs. Participants engaged in computer-based MOBA or FPS video games, using a computer mouse and keyboard. The mouse operation was conducted with the right hand, and the mouse sensitivity was set between 400 and 3000 dots per inch (DPI). Participants were excluded if they reported acute/chronic upper body musculoskeletal disorders, severe migraines, epilepsy, or significant physical or cognitive stress on the day before [19]. Participants were recruited through social media (*Discord*, *Instagram*, *LinkedIn*), in person at gaming venues and universities in Cologne, and via esports organizations. Recruitment was open to individuals of all gender identities; however, biological sex was also recorded and used in the analysis due to the physiological focus of the study.

### Procedure

**Fig. 2:**
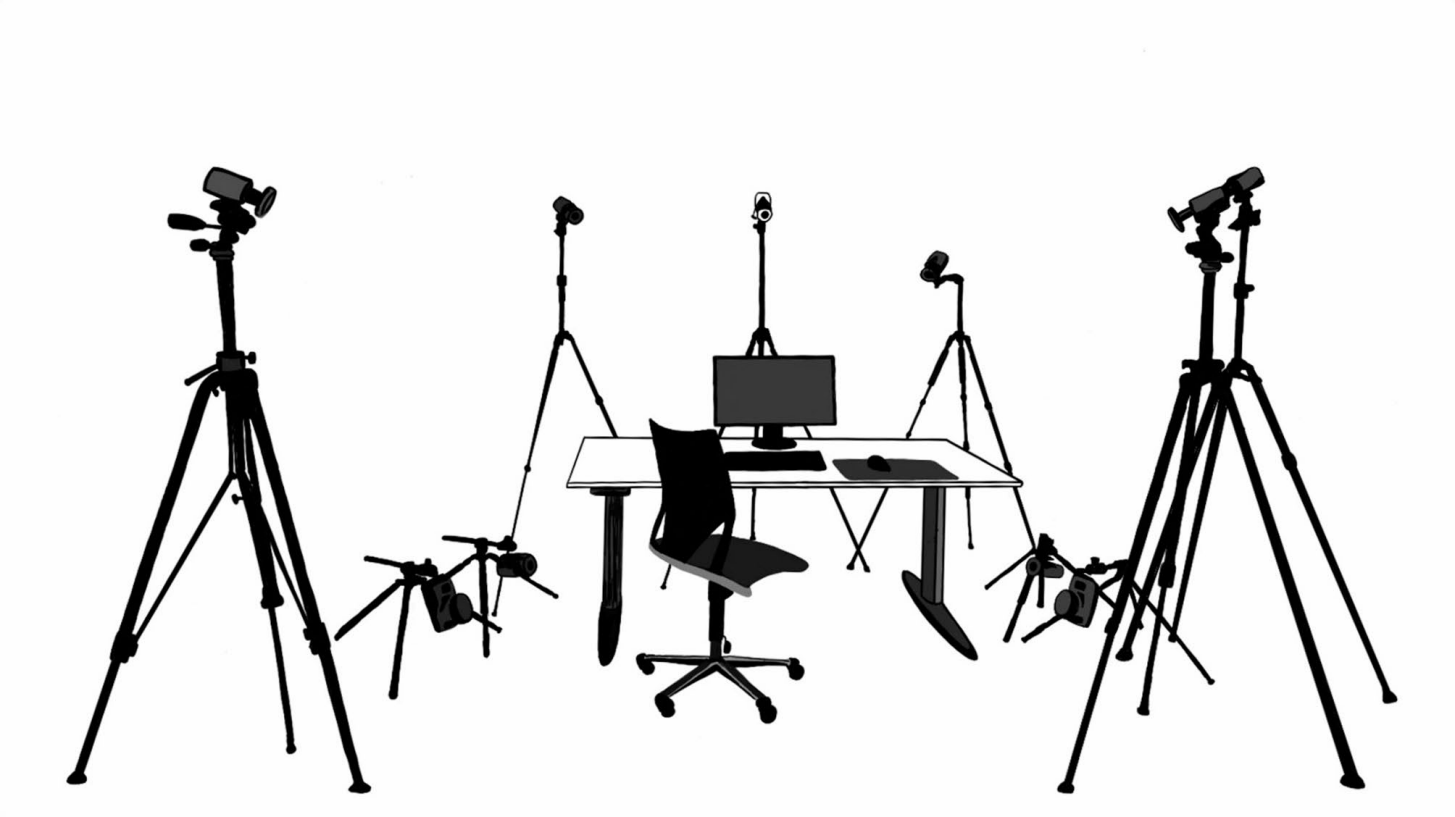
Standardized testing station with 10 motion capture cameras

At the beginning of the examination, eligibility was verified, and anthropometric data were collected. Participants were instructed to refrain from cognitively or physically demanding activities on both the day prior to and the day of testing. Additionally, they were asked to abstain from alcohol for at least 12 h, avoid caffeinated beverages for five hours, and not to apply any lotions or creams on the day of the test. For electromyographic (EMG) analysis, the extensor digitorum communis and trapezius descendens were prepared according to *SENIAM* guidelines [35]. Passive reflective markers were then placed on the upper body and upper limb for kinematic data recording. Participants were asked to complete a partially standardized online questionnaire at the testing station to collect demographic data, health status, physical activity, musculoskeletal disorders and video gaming behavior. Physical activity was assessed with the *European Health Interview Survey - Physical Activity Questionnaire* (EHIS-PAQ) [36]. A comprehensive description of the questionnaire used in the current study is available in our previous publication [19]. The testing station consisted of an adjustable chair without armrests to allow for better hip motion capture and no headrest, an adjustable desk, ten motion capture cameras (*Miqus M3* & *Oqus 100*, *Qualysis*) and a provided gaming setup described in detail in the appendix. To standardize the parameters of video game exposure the participants were not allowed to play on personal hardware. Fig. 2 shows the standardized testing station. Additional photo material of the real experimental setup can be found in the appendix (Supplementary Figs. 12–13).

Prior to the start of the measurement, participants were asked to do their best to win the games. Subjects were required to play ranked matches on their primary accounts in a self-selected MOBA (*League of Legends*,* Defense of the Ancients 2*) or FPS (*Counter-Strike*,* Valorant*,* Overwatch*,* Rainbow Six Siege*) video game, to simulate typical tournament stress conditions. Internet connectivity was stable throughout all sessions. No latency issues or disconnections were observed by the research team, and none of the participants reported any connectivity-related disturbances during gameplay. EMG and kinematic data were recorded continuously during the competitive video gaming sessions and saved in 15-minute intervals. To meet the 90-minute minimum for data collection, participants had to play multiple rounds of the game, which typically lasted 25–45 min. Depending on the length of each game, sessions ranged from 90 to 120 min. After 120 min data recordings were stopped. Competitive video gaming sessions were interrupted by a 10-minute passive sitting [19], which reflects the average break between tournament games [37–39]. During the break, the consumption of alcoholic or caffeinated beverages and smoking was explicitly prohibited, with no other restrictions on food or caloric intake. A 5-minute passive sedentary recovery period was included in the study after the second competitive video game session.

### Data collection & processing

**Fig. 3:**
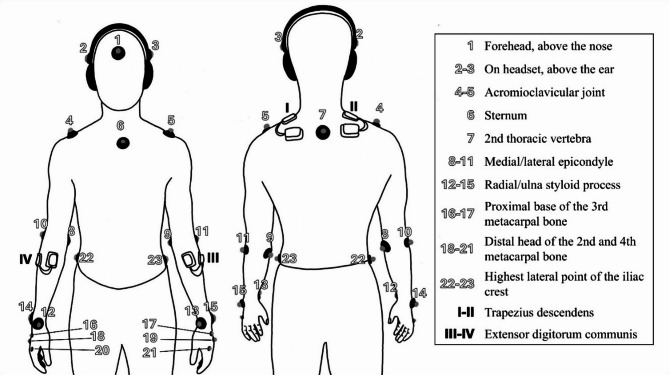
Marker and sensor placement

EMG data was collected at 2000 Hz with a wireless system (*Ultium*,* Noraxon*) [40]. Four dual Ag/AgCl electrodes with an interelectrode distance of 20 mm were placed on the trapezius descendens and the extensor digitorum communis (Fig. 3). Due to the high probability of cross-talk in the analysis of the extensor digitorum [41], the term wrist extensors will be used in the remainder of this article. The raw data was bandpass (4th order recursive Butterworth, 4–450 Hz) and notch filtered (50 Hz) to eliminate power line interference. The EMG amplitude or the Electrical Activity (EA) was calculated by summing up the absolute values of the root mean square (RMS) samples for 10-s periods [42]. RMS values were normalized to isometric maximal voluntary contraction (MVC), in accordance with established guidelines [43]. Each muscle underwent three isometric MVC trials, which last five seconds, with about 30 s rest intervals between attempts [15, 44]. The EMG signal was segmented into 400 ms moving windows, RMS values were computed for each window, and the peak RMS value from each trial was identified. The highest value recorded across the three trials was used for normalization. The evaluation of both muscle groups was conducted in a seated position. For upper trapezius testing, participants were instructed to perform an isometric shoulder shrug in a neutral shoulder position while holding a strapped grip with the arm extended. Wrist extensor testing was performed with the forearm placed on an elevated surface, the shoulder in a neutral position, and the wrist maintained in approximately 20° of extension. The participants were instructed to extend their back of the hand against a fixed strap. This wrist posture was chosen to approximate the muscle’s optimal length on the length–tension curve and to replicate wrist positioning commonly observed esports participation [44, 45]. Prior to MVC testing, participants completed a brief standardized warm-up to activate the relevant muscles without inducing fatigue. The warm-up protocol consisted of 20 repetitions of wrist and shoulder circumductions in both directions, followed by dynamic stretching exercises: ten repetitions of wrist flexion and extension, and ten lateral dynamic stretches targeting the upper trapezius on each side. To analyze the power spectrum of EMG the median frequency (MDF) was calculated using Fast Fourier Transformation for 10-s periods [42]. MDF and RMS were visually inspected for artefacts. Non-physiological artifacts were spline interpolated [46].

Kinematic data was collected at 150 Hz with a marker based mocap system (*Qualisys Track Manager*, version 2020.2) [47]. Twenty-three passive reflective markers were attached to the participants using an adapted version of the *Qualisys* sports marker set (Fig. 3) [48]. Markers on the head, torso, and upper limbs were included as required by the kinematic model and may be utilized in subsequent analyses. No additional markers were attached to the gaming equipment or the testing station. Recording gaps of up to 30 frames were automatically filled using polynomial methods, while larger gaps were filled with relational rigid body gap-filling in *Qualisys Track Manager* [47].

### Outcomes

To observe muscular fatigue, the EMG amplitude and power spectrum were analyzed. In particular, a temporal downshift in the power spectrum is commonly associated with muscular fatigue [49]. To ensure greater validity, the EMG amplitude was also analyzed, as a simultaneous increase in amplitude is indicative of muscle fatigue as well [42, 50].

The analysis of the kinematic data was informed by a previous study in order to ensure greater comparability [31]. The primary outcome parameters were: (1) the area of displacement (AoD), defined by an ellipse representing 95% of the mouse movement within the x-y axes on the table; (2) the cumulative distance traveled by the mouse hand during the different competitive games; and (3) the number of velocity zero-crossings of the mouse hand along the x-axes. Additionally, the time spent in pre-defined wrist joint angles was analyzed to identify potentially harmful positions, as defined by the Institute for Research and Testing of the German Social Accident Insurance [51]. Due to the lack of a standardized starting position, the respective mean wrist joint angle for each participant was used as the neutral position (0°).

Furthermore, an interaction analysis was conducted to investigate the effect of mouse sensitivity on kinematic data. Therefore, the “effective DPI” (eDPI), derived from the product DPI and of ingame mouse sensitivity, was calculated and z-transformed to facilitate comparisons across video game genres.

### Statistical methods

The statistical analysis was conducted using the *RStudio* software (version 4.3.1) [52]. Due to the variable length of competitive video gaming sessions, only the first (_1) and the last game (_2) of each session were analyzed. These analyses include the first and last 5 min of each respective game. Single extreme outliers were excluded if they exceeded or fell below three times the interquartile range [53]. Descriptive statistics are presented, including the mean and standard deviation (SD) values. Baseline group differences were evaluated based on the distribution and scale of the variables. Continuous variables were tested using either an *unpaired t-test* or the *Wilcoxon rank-sum test*, depending on normality and variance assumptions. Categorical variables were analyzed using *Fisher’s exact test*.

For the ANOVAs, normality at each measurement time for each variable and group was visually assessed using quantile-quantile (QQ) plots. Even if normal distribution is violated, the ANOVA remains robust [54, 55]. QQ plots for each variable are included in the appendix (Supplementary Figs. 1–11). In addition, homogeneity of variances was checked visually and homogeneity of covariances with the *Box’s M-Test* for the mixed ANOVA. If either of these requirements were violated, a robust mixed ANOVA was used. Sphericity was tested using *Mauchly’s test*, and if the assumption was violated (*p* ≤ 0.05), the *Greenhouse-Geisser* (ε < 0.75) or *Huynh-Feldt* (ε ≥ 0.75) correction was applied [56]. Changes over time were tested with *Bonferroni* post-hoc analysis. Differences between groups were evaluated with the independent *t-Test*. Effect sizes were calculated by using *Cohen’s d* and interpreted as small = 0.2, moderate = 0.5 and large = 0.8 effect [57]. The interaction with z-transformed eDPI was analyzed using robust regression analysis with MM-estimators, as assumptions of the standard model were violated. The significance level for all analyses was set at *p* < 0.05. In line with the open science principle, all data as well as the R-syntax will be available after one year of publication.

## Results

### Participants

Table 1 displays the sample characteristics and baseline differences. A total of 32 male participants, predominantly right-handed (91%), with a mean age of 23.8 years (± 3.4), were included in the study, with no dropouts. The majority (69%) of participants were currently college students and held at least an A-Level degree (85%). On average, participants exhibited a BMI of 24.8 kg/m² (± 3.7). The mean weekly physical activity level among participants was 307.8 min/week (± 3.4). On average participants played video games 3.6 h/day (± 2.0) and had 12 years (± 4.3) of video game experience. The dominant video game genre was MOBA with 69%. The majority of participants (59%) achieved rankings within the top 5%, with MOBA players more frequently represented in higher competitive tiers than FPS players (*p* = 0.025). Additionally, the prevalence of musculoskeletal disorders is reported in detail in our previous study [19]. In that study, wrist and hand discomfort within the seven days prior to measurement was the most commonly reported issue (12.5%). Throughout the quasi-experimental study, no significant changes in discomfort or pain were observed [19].

**Table 1:** Sample characteristics and baseline differences

	FPS (*n* = 10)	MOBA (*n* = 22)	*p*-value
**Age** [years]	24.9 ± 4.28	23.3 ± 2.82	0.473^w^
**Height** [cm]	181 ± 5.33	180 ± 7.32	0.610^w^
**Body mass** [kg]	76.1 ± 10.3	83 ± 15	0.198^t^
**BMI** [kg/m²]	23.1 ± 2.54	25.6 ± 3.9	0.064^w^
**BMI categories**, n (%)			0.305^f^
Underweight	0 (0%)	0 (0%)	
Normal weight	8 (80%)	11 (50%)	
Overweight	2 (20%)	9 (40.9%)	
Obesity	0 (0%)	2 (9.1%)	
**Total Physical Activity** [min/week]	267.5 ± 173.1	326.14 ± 380.44	0.728^w^
> 150 min, n (%)	7 (70%)	13 (59.1%)	0.703^f^
> 300 min, n (%)	2 (20%)	10 (45.5%)	0.248^f^
MSTW, n (%)	8 (80%)	9 (40.9%)	0.061^f^
**Video game** **playtime** [h/day]	3.39 ± 1.89	3.7 ± 2.01	0.597^w^
**Video game** **experience** [years]	12 ± 4.9	12.9 ± 4.03	0.198^w^
**Handedness**, n (%)			0.090^w^
Left	0 (0%)	1 (4.6%)	
Right	8 (80%)	21 (95.5%)	
Both	2 (20%)	0 (0%)	
**In-game rank** **Distribution**, n (%)			**0.0247** ^**w**^
1%	1 (10%)	8 (36.4%)	
5%	2 (20%)	8 (36.4%)	
10%	5 (50%)	1 (4.6%)	
20%	2 (20%)	5 (22.7%)	

FPS: first person shooter; MSTW: muscle strengthening twice a week; MOBA: multiplayer online battle arena; ^f^ Fisher’s exact test; ^t^ t-test; ^w^ Wilcoxon rank-sum test; **Bold** = significant

### Muscular fatigue

#### Median frequency

**Fig. 4:**
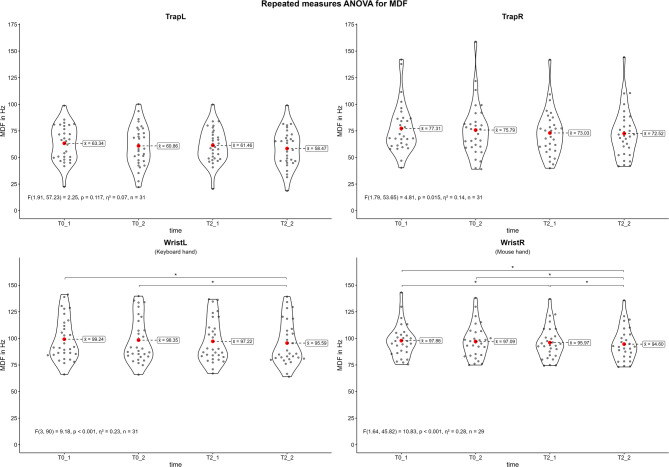
Violin plots of the median frequency (MDF) during the competitive video gaming sessions. Trap = upper trapezius; Wrist = wrist extensor; L = left side; R = right side; T0 = first competitive session, T2 = second competitive session, _1 = first game, _2 = last game; *: *p* ≤ 0.05

Fig. 4 shows the violin plots for the MDF of the recorded muscles during the competitive video gaming sessions. The results of the repeated measures ANOVA indicate significant differences in the MDF of the right trapezius (*p* = 0.015, η² = 0.14), the left wrist extensors (*p* < 0.001, η² = 0.23) and the right wrist extensors (*p* < 0.001, η² = 0.28). The post-hoc tests revealed that the MDF of the right trapezius did not differ between measurements. In contrast, both wrist extensors showed significant changes between the first and last (T0_1 vs. T2_2), as well as the second and last measurement (T0_2 vs. T2_2). Overall, there was a decrease in MDF over time. The MDF of the left wrist extensor decreased from 99.24 Hz (T0_1) to 95.59 Hz (T2_2) (-3.7%). The MDF of the right wrist extensor decreased from 97.86 Hz (T0_1) to 94.60 Hz (T2_2) (-3.3%). The effect sizes of all significant changes can be considered moderate. The results of all post-hoc tests are shown in the appendix (Supplementary Tables 2–4).

#### Electric amplitude

**Fig. 5:**
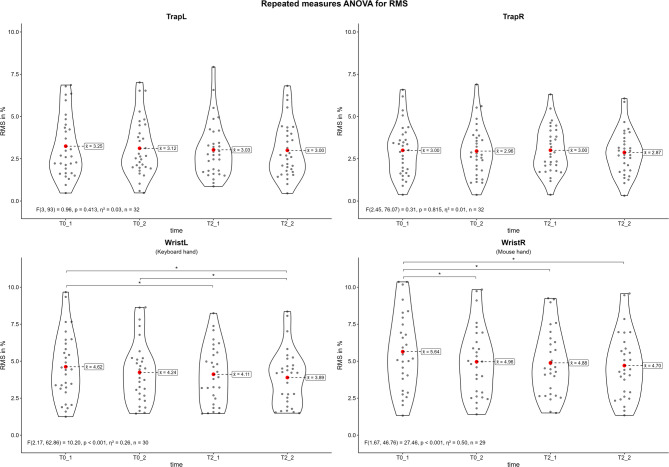
Violin plots of the root mean square (RMS) during the competitive video gaming sessions. Trap = upper trapezius; Wrist = wrist extensor; L = left side; R = right side; T0 = first competitive session, T2 = second competitive session, _1 = first game, _2 = last game; *: *p* ≤ 0.05

Fig. 5 shows the violin plots for the normalized RMS of the different muscles during the competitive video gaming sessions. The results of the repeated measures ANOVA indicate significant differences in the normalized RMS of both wrist extensors (left: *p* < 0.001, η² = 0.26; right: *p* < 0.001, η² = 0.50). The post-hoc tests revealed significant differences between the first and the last (T0_1 vs. T2_2) and the first and the third measurement (T0_1 vs. T2_1). Additionally, the left wrist extensor showed a significant difference between the second and final measurements (T0_2 vs. T2_2), whereas the right wrist extensor showed a significant difference between the first and second measurements (T0_1 vs. T0_2). Overall, there was a decrease in EA over time. The normalized RMS of the left wrist extensor decreased from average 4.62% (T0_1) to 3.89% (T2_2) (-15.8%) with moderate effect sizes. The normalized RMS of the right wrist extensor decreased from 5.64% (T0_1) to 4.70% (T2_2) (-16.6%) with large effect sizes. The results of all post-hoc tests are shown in the appendix (Supplementary Tables 5–6).

### Kinematic data

#### Wrist angles

Table 2 presents the percentage of time the mouse hand spent in various wrist angles in radial or ulnar adduction across different video game genres and measurement times. In both video game genres and across all measurement times, the first range of joint angle positions (-10° to 10°) are predominantly utilized, accounting for over 95% of the time. This zone is classified as the neutral zone [51]. FPS players spent less time in the first range of joint angle positions compared to MOBA players across measurements. Consequently, FPS players allocated more time to the second and third joint angle positions, except for the second measurement (T0_2).

**Table 2:** Percentage of time mouse hand spent in radial or ulnar adduction by genre and time

	FPS (*n* = 10)			MOBA (*n* = 22)		
	-10° to 10°	-10° to -25° and 10° to 15°	< -25° and >15°	-10° to 10°	-10° to -25° and 10° to 15°	< -25° and >15°
T0_1	95.95%	2.35%	1.70%	99.44%	0.36%	0.20%
T0_2	97.24%	1.99%	0.77%	98.04%	0.69%	1.26%
T2_1	96.41%	2.13%	1.46%	98.65%	0.81%	0.54%
T2_2	95.82%	1.95%	2.23%	97.20%	1.86%	0.94%

FPS: first person shooter; MOBA: multiplayer online battle arena. Positive degrees = radial adduction; Negative degrees = ulnar adduction

#### Cumulative distance

Table 3 shows the descriptive and inferential statistics of the cumulative distance across video game genres and measurement times. Overall, the cumulative distance decreased over time in FPS players from the first to the fourth measurement (-21.49%). In contrast, for MOBA players, it initially decreased to the third measurement (-11.92%) before increasing at the fourth measurement (+8.51%). The robust mixed ANOVA showed no significant interaction effect (Q(3, 4.58) = 1.80, *p* = 0.272). Similarly, no significant within-subject effect for time was observed (Q(3, 4.58) = 1.02, *p* = 0.463). However, a significant between-subject effect for the video game genre was identified (Q(1, 5.29) = 18.94, *p* = 0.006). In particular, FPS players exhibited significantly greater mean cumulative distance compared to MOBA players.

**Table 3:** Robust mixed ANOVA for cumulative distance [m]

	FPS (*n* = 10)		MOBA (*n* = 22)	
	M	SD	M	SD
T0_1	63.34	39.45	19.03	8.44
T0_2	55.10	32.72	19.48	10.85
T2_1	55.05	32.24	16.76	7.01
T2_2	49.73	34.66	20.65	7.84
**ANOVA Results**				
*Group*	Q(1, 5.29) = 18.94, ***p* = 0.006**			
*Time*	Q(3, 4.58) = 1.02, *p* = 0.463			
*Interaction*	Q(3, 4.58) = 1.80, *p* = 0.272			

FPS: first person shooter; MOBA: multiplayer online battle arena

#### Area of displacement

Table 4 presents the descriptive and inferential statistics for the AoD across video game genres and measurement times. Overall, the AoD increased from the first to the last measurement in both FPS (+ 33.13%) and MOBA (+ 18.69%) players, with notable differences observed between the two groups. The robust mixed ANOVA showed no significant interaction effect Q(3, 5.65) = 0.65, *p* = 0.61). Furthermore, neither the within-subject effect (Q(3, 5.65) = 0.91, *p* = 0.49) nor the between-subject effect Q(1, 5.47) = 5.58, *p* = 0.06) was statistically significant.

**Table 4:** Robust mixed ANOVA for area of displacement [cm²]

	FPS (*n* = 10)		MOBA (*n* = 22)	
	M	SD	M	SD
T0_1	68.66	54.91	32.05	23.67
T0_2	85.59	58.85	32.99	27.24
T2_1	83.07	48.76	34.20	30.96
T2_2	91.41	74.05	38.04	30.15
**ANOVA Results**				
*Group*	Q(1, 5.47) = 5.58, ***p*** = **0.06**			
*Time*	Q(3, 5.65) = 0.91, *p* = 0.49			
*Interaction*	Q(3, 5.65) = 0.65, *p* = 0.61			

FPS: first person shooter; MOBA: multiplayer online battle arena

#### Zero-crossings

Table 5 shows the descriptive and inferential statistics for total number velocity zero-crossings across video game genres and measurement times. Overall, the number of zero-crossings decreased from the first to the third measurement in both FPS players (-16.75%) and MOBA players (-9.57%). At the fourth measurement, the zero-crossings increased again, exceeding the baseline value in FPS players (101.03%) and approaching it in MOBA players (97.14%). The mixed ANOVA showed no significant interaction effect (F(3, 87) = 4.46, *p* = 0.713, pes = 0.016). Similarly, no significant within-subject effect for time was observed (F(3, 87) = 1.36, *p* = 0.26, pes = 0.045). However, a significant between-subject effect for the video game genre was identified (F(1, 29) = 4.47, *p* = 0.043, pes = 0.134). Specifically, FPS players exhibited a significantly higher total number of velocity zero-crossings on average compared to MOBA players.

**Table 5:** Mixed ANOVA for total number velocity zero-crossings

	FPS (*n* = 10)		MOBA (*n* = 22)	
	M	SD	M	SD
T0_1	1167.00	476.98	773.62	546.90
T0_2	994.50	356.45	723.71	485.24
T2_1	971.50	494.27	699.57	532.37
T2_2	1179.00	431.06	751.57	526.19
**ANOVA Results**				
*Group*	F(1, 29) = 4.47, ***p*** = **0.043**, pes = 0.134			
*Time*	F(3, 87) = 1.36, *p* = 0.26, pes = 0.045			
*Interaction*	F(3, 87) = 4.46, *p* = 0.713, pes = 0.016			

FPS: first person shooter; MOBA: multiplayer online battle arena

#### Interaction eDPI

The robust regression analysis revealed statistically significant negative correlations between z-transformed eDPI and cumulative distance (β = −5.02, SE = 1.39, t = − 3.60, *p* < 0.001) and AoD (β = −9.29, SE = 2.20, t = − 4.22, *p* < 0.001). This indicates that for each one-unit increase in eDPI, the cumulative distance decreased by approximately 5.02 cm and the AoD decreased by approximately 9.29 cm². No statistically significant relationship was observed between the number of zero-crossings and eDPI (β = −60.49, SE = 48.23, t = − 1.25, *p* = 0.212).

## Discussion

The objective of this study was to examine the impact of competitive video gaming on muscular fatigue and wrist kinematics. The main findings partially support the primary hypothesis indicating that muscular fatigue increased over time in wrist extensors, as evidenced by a significant downshift in the EMG frequency spectrum. Atypically, this was accompanied by an RMS decrease. However, wrist kinematics differed only between video game genres but not across time or in interaction. On average, FPS players exhibited greater cumulative distances and higher numbers of velocity zero-crossings, and they also tended to cover larger AoDs. Notably, a 10-minute passive break between competitive gaming sessions did not effectively reduce physical load.

### Muscular fatigue

Only wrist extensors demonstrated a significant decrease in MDF and RMS over time (Figs. 4 and 5). The MDF decreased about 3.7% in the left and 3.3% in right wrist extensors with a moderate effect size. This temporal downshift in the power spectrum is commonly associated with muscular fatigue [49]. This is plausible because of the nature of competitive video gaming and the involvement of the hands to interact with the virtual environment [6]. Similar findings are demonstrated in other video gaming exposure studies were smartphone gaming decreased MDF in the erector spinae [26] or extensor and abductor pollicis brevis [27]. Related effects can be found after prolonged keyboard typing [15] or piano tasks [9]. In contrast the trapezius descendants demonstrated a non-significant decrease of 7.7% on the left and 6.2% on the right side. The reasons for this may be a less involved activity compared to the hands or a higher variability between subjects indicated by a larger interquartile range (Fig. 2). Controverse findings where demonstrated after 40 min of sedentary computer work where the MDF decreased significantly in the upper trapezius [28]. In contrast to this and in line with current findings, smartphone gaming did not decreased MDF significantly [26].

The RMS was reduced by 15.8% in the left and 16.6% in right wrist extensors with large effect sizes. This contrasts other findings, which demonstrated muscular fatigue in a simultaneous decrease in MDF and increase in RMS according to the joint analysis of the spectral and amplitude (JASA) [28, 42]. The JASA method is based on the association between muscular force production and fatigue state, as well as the EMG amplitude and spectrum. Controverse effects were observed in similar studies with low-effort repetitive activities with an increase [9, 15, 28] or decrease [16, 58] in RMS over time. However, EMG amplitude is a highly task-dependent parameter. In high-intensity or maximal contractions, EMG amplitude typically increases over time due to the recruitment of additional motor units (MUs) to maintain the required force output, thereby compensating for fatigue in already active fibers [42, 59]. A similar trend has been observed in sustained submaximal contractions [9, 15]. JASA is based on the same principle of constant-force contractions [42], which may not reflect the load characteristics and intermittent nature of competitive video gaming. In contrast, intermittent, low-effort activities, may exhibit a decreases in EMG amplitude over time [16, 60]. Several mechanisms have been proposed in the literature to explain this observation, including limited recruitment of additional MUs, potentiation of muscle fibers, or MU rotation. It has been hypothesized that low-force tasks primarily activate only a small subset of the available MUs, which are predominantly slow-twitch (type I) fibers, often referred to as “Cinderella fibers” due to their continuous activation during low-intensity, sustained activities [61]. These motor units remain active until complete muscle relaxation, making them particularly susceptible to overuse and fatigue-related strain. Furthermore, the decline in EMG amplitude may be attributed to muscle fiber potentiation, whereby the muscle produces greater force per stimulus at a given level of neural input [62]. Potentiation is regarded as an initial phase in the progression of muscle fatigue. It is hypothesized that this phenomenon functions as a protective mechanism by enhancing muscular efficiency, that is, by increasing the force output per unit of neural input [16]. Another possible mechanism at low contraction levels is MU rotation [63, 64]. In this process, fatigued MUs temporarily deactivate while fresh MUs are recruited to sustain force output. If the newly recruited MUs generate less electrical activity, such as smaller or less active units, or if the net effect of this rotation does not lead to an increase in EMG amplitude, the RMS value may remain stable or even decline [64]. These neuromuscular adaptations, such as selective MU recruitment, potentiation, and rotation, can be more accurately characterized using high-density EMG (HD-EMG), which provides spatial resolution of MU activity [65]. Therefore, future studies investigating the underlying motor unit behavior during low-intensity, intermittent tasks, such as competitive gaming, should consider employing HD-EMG.

These physiological mechanisms may offer a potential explanation for the observed decline in EMG amplitude during low-effort, intermittent activities such as competitive video gaming. Notably, the nature of esports includes dynamic and irregular task demands that differ from standardized fatigue protocols. For example, esports athletes often experience brief periods of reduced muscular activity, such as during in-game respawns, between rounds, or while waiting in matchmaking queues, that allow partial recovery [19]. These intermittent recovery phases could reduce continuous neuromuscular loading, potentially influencing fatigue development and masking typical EMG fatigue patterns [16]. Therefore, the specific structural features of competitive video gaming may contribute to the deviation from traditional EMG fatigue signatures observed in constant-load tasks.

### Wrist kinematics

FPS players move their mouse hands over significantly greater distances and exhibit higher numbers of zero-crossings (lateral movements of the wrist) than MOBA players (Tables 3 and 5). Additionally, FPS players tend to cover larger areas than MOBA players, although this difference was not statistically significant (Table 4). However, no effect of time was observed, which partially confirms the second hypothesis. The group differences may be explained by the use of lower mouse sensitivity settings in the FPS group, as FPS games require more precise movements than MOBA games [29]. Consequently, FPS players have to move their upper extremities more [31]. A negative relationship between eDPI and cumulative distances, as well as AoD, was observed in the current study. Therefore, it can be indirectly concluded that FPS players use lower mouse sensitivity settings, confirming the sub-hypothesis. These findings suggest that the greater movement observed in FPS players is primarily performance-driven, resulting from genre-specific motor demands and strategic use of lower sensitivity settings to improve aiming precision. Furthermore, the current study demonstrated a significant difference in zero-crossings between FPS and MOBA player which is in contrast to other findings [31]. Additionally, the number of zero-crossings observed in the current study was substantially lower than in that prior study, where both FPS and MOBA players reached approximately 2,250 zero-crossings over a 10-minute period. One potential explanation for this discrepancy is the use of different measurement tools. The present study employed passive optical motion capture, whereas Dupuy et al. [31] used inertial measurement units, which may result in divergent sensitivity to movement characteristics. Additionally, a baseline difference in rank distribution was observed, with MOBA players more frequently represented in higher competitive tiers than FPS players, which could have influenced the group differences observed in the current study.

The presented kinematic data indicate continuous, highly repetitive, and large-scale movements over 3–4 h of competitive video gaming, with differences between FPS and MOBA players. This behavior could increase the risk of muscular fatigue [28]. In consideration of the present findings, this could explain why the wrist extensors fatigued significantly. Conversely, the upper trapezius only showed tendencies of muscular fatigue. In addition, a 10-minute passive break between competitive gaming sessions did not affect either muscular fatigue or kinematic parameters. Based on the current evidence break patterns should be more physically active to decrease muscular fatigue and perceived discomfort [28] and physical exertion [19]. Furthermore, active breaks could increase cognitive functions and consequently esports performance [66–68]. Additionally, the current study highlighted that even a single competitive video gaming session of 3–4 h increased muscular fatigue significantly. Considering the daily training habits of esports athletes, which range between 4 and 10 h/day [4, 5], this could contribute to MSDs in the long term [21]. Therefore, it is highly recommended for practitioners to not only schedule breaks, but also to plan specific break behaviors that include physical activity [69]. For an even greater preventive effect, regular exercise can prevent premature muscular fatigue and MSDs [6, 21].

### Limitations

The results of this study must be interpreted in light of several limitations. First, the interpretation of EMG-based fatigue markers may be influenced by the structure of competitive gaming sessions. Participants experienced passive breaks while waiting in matchmaking queues, which sometimes lasted up to 10 min depending on in-game rank. These intermittent recovery periods, which reflecting real-world tournament conditions [37–39], may have attenuated muscle fatigue and contributed to the atypical RMS trends observed. Additionally, EMG analysis was conducted in the absence of force or task performance data, which limited the ability to link electrophysiological changes directly to functional fatigue. Potential EMG crosstalk between closely spaced wrist extensor muscles is another possible limitation, as it may confound muscle-specific signal interpretation [41]. Second, the absence of a standardized starting wrist position may have introduced variability in kinematic outcomes, particularly in zero-crossing analyses. Consequently, absolute joint angle measurements may have varied between individuals, potentially affecting the comparability of the kinematic data. Furthermore, two participants were classified as obese (BMI ≥ 30), which may have influenced individual neuromuscular or biomechanical responses. No subgroup analysis was conducted due to the small number.

Beyond these limitations, several methodological choices should also be acknowledged. The present study included only male esports athletes, reflecting current gender distributions in esports but limiting generalizability [5]. Future research should aim for more gender-diverse samples. The study did not include a control group, and randomization was not feasible, limiting causal inference. Finally, the distribution of group sizes was imbalanced (MOBA: *n* = 22; FPS: *n* = 10) due to the consecutive inclusion of participants based on their eligibility rather than stratification by game genre. This imbalance may have influenced the outcomes of between-group comparisons.

## Conclusion

In summary, competitive video gaming for 3–4 h may induce muscular fatigue in esports athletes. Additionally, FPS players demonstrated significantly greater cumulative distances exhibited higher numbers of lateral wrist movements, and tended to cover larger AoDs than MOBA players. No effect of time was found for kinematic parameter. A passive break did not provide sufficient short-term regeneration for the observed parameters. Without additional physical activity or exercises, these physical demands could lead to higher risks of MSDs and consequently to earlier retirements for esports athletes. Practically, esports athletes are advised to regularly monitor their training habits, particularly their break schedules. Further research should evaluate different methods for analyzing muscular fatigue in esports, with particular emphasis on wavelet transformation. Additionally, different break activities should be compared to evaluate the best opportunities to reduce and avoid muscular fatigue, but also to enhance performance of esports athletes.

## Supplementary Information

Below is the link to the electronic supplementary material.

Supplementary Material 1

## Data Availability

The raw data supporting the conclusions of this article will be made available by the authors one year after publication. All data and materials from this study will be available on the *Open Science Framework* ([https://osf.io/zhy29/](https://osf.io/zhy29/)).

## Abbreviations

AoD: Area of displacement
APM: Actions per minute
DPI: Dots per inch
eDPI: Effective dots per inch
Esports: Electronic sports
EMG: Electromyography
FPS: First-person shooter
HD-EMG: High-density electromyography
JASA: Joint analysis of the spectral and amplitude
MDF: Median frequency
MOBA: Multiplayer online battle arena
MSD: Musculoskeletal disorders
MVC: Maximal voluntary contraction
RMS: Root mean square
QQ plot: Quantile-quantile plot
