# Supplementary material for: Wrist extensor fatigue and game-genre-specific kinematic changes in esports athletes: a quasi-experimental study
Source: BMC Sports Sci Med Rehabil. 2025 Sep 1;17:261. doi: 10.1186/s13102-025-01305-0 (PMC12400618; doi:10.1186/s13102-025-01305-0)
Supplement: Supplementary file 1 — Supplementary Material 1 [file 13102_2025_1305_MOESM1_ESM.pdf]

Supplementary Material

Wrist extensor fatigue and game-genre-specific kinematic changes in esports athletes: a quasi-experimental study

1 Supplementary Data

The raw data supporting the conclusions of this article will be made available by the authors one year after publication. All data and materials from this study are available on the *Open Science Framework* (DOI: 10.17605/OSF.IO/ZHY29, https://osf.io/zhy29/).

2 Supplementary Figures and Tables

2.1 Supplementary Figures

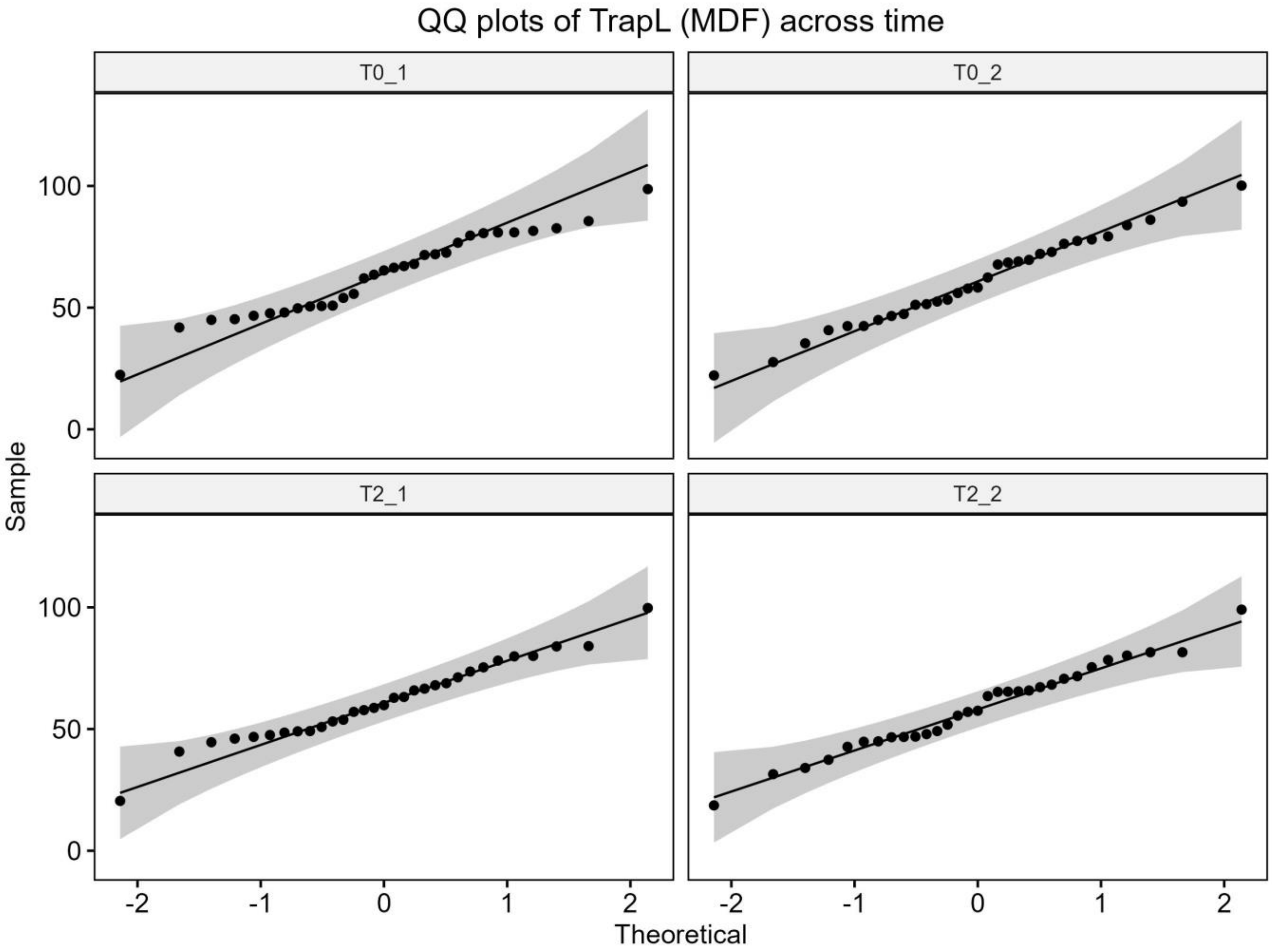

Supplementary Figure 1: Normal distribution of median frequency (MDF) of the left upper trapezius (TrapL) at measurement times. T0 = first competitive session, T2 = second competitive session, _1 = first game, _2 = last game.

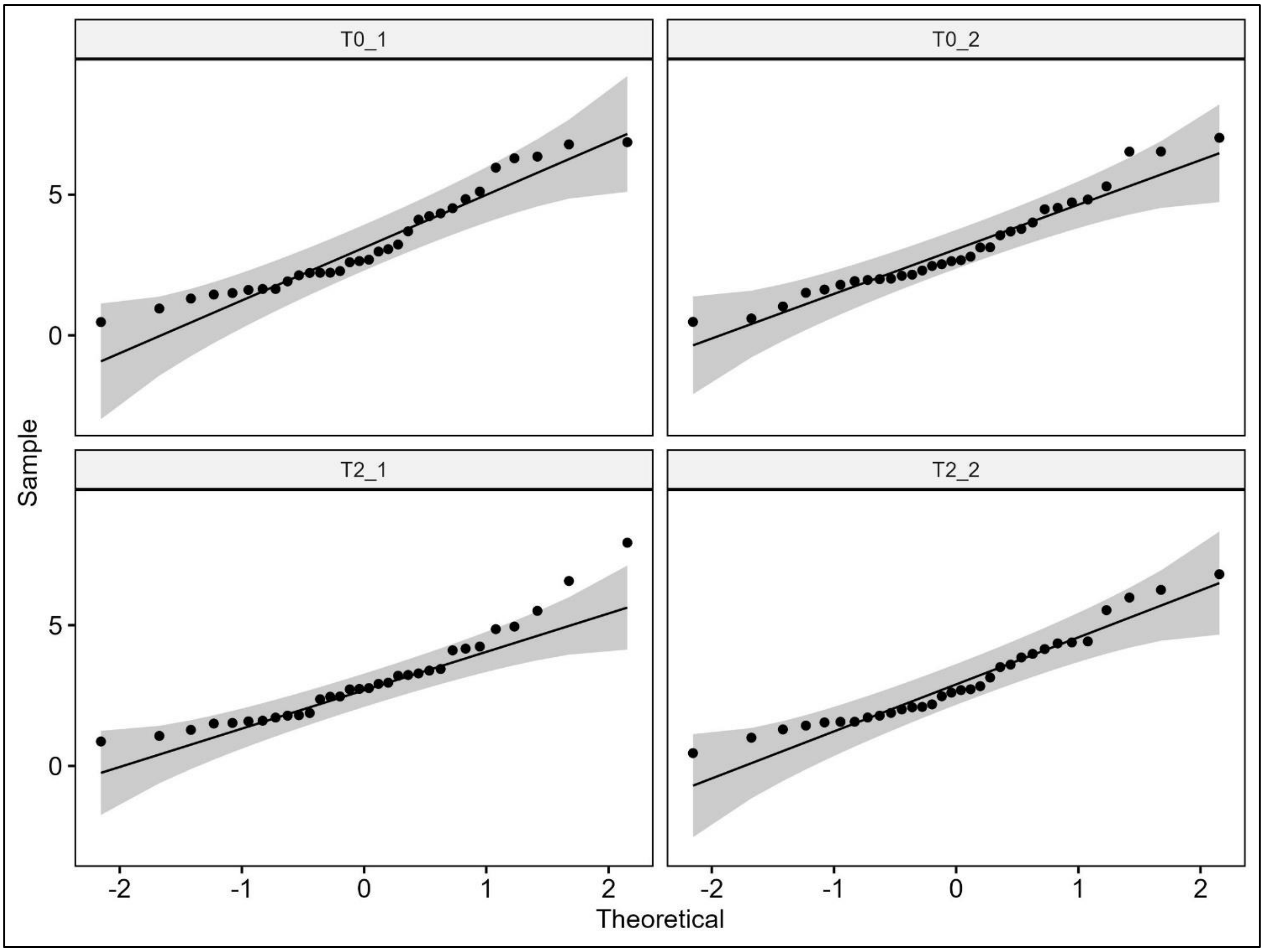

Supplementary Figure 2: Normal distribution of root mean square (RMS) of the left upper trapezius (TrapL) at measurement times. T0 = first competitive session, T2 = second competitive session, _1 = first game, _2 = last game.

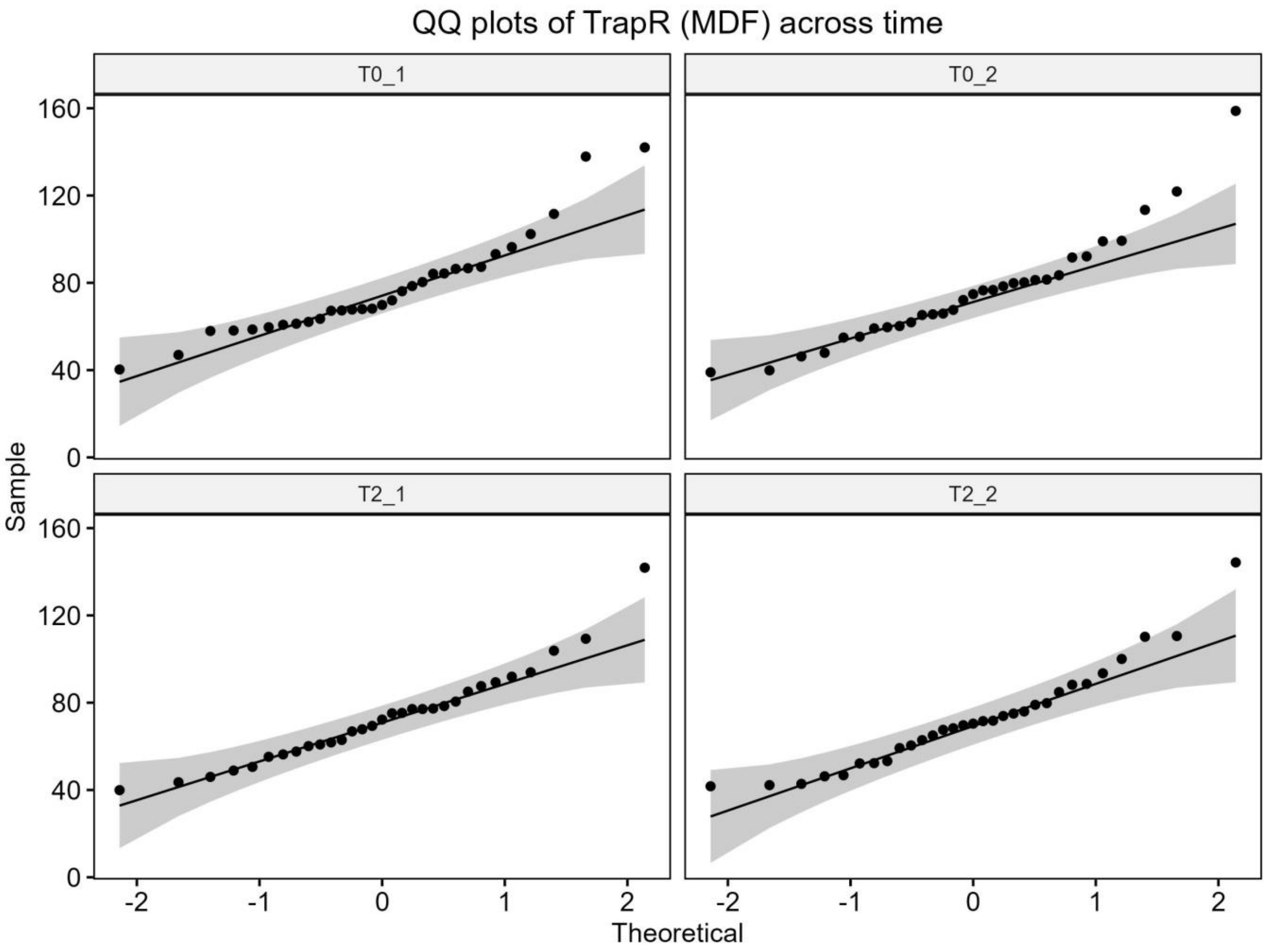

Supplementary Figure 3: Normal distribution of median frequency (MDF) of the right upper trapezius (TrapR) at measurement times. T0 = first competitive session, T2 = second competitive session, _1 = first game, _2 = last game.

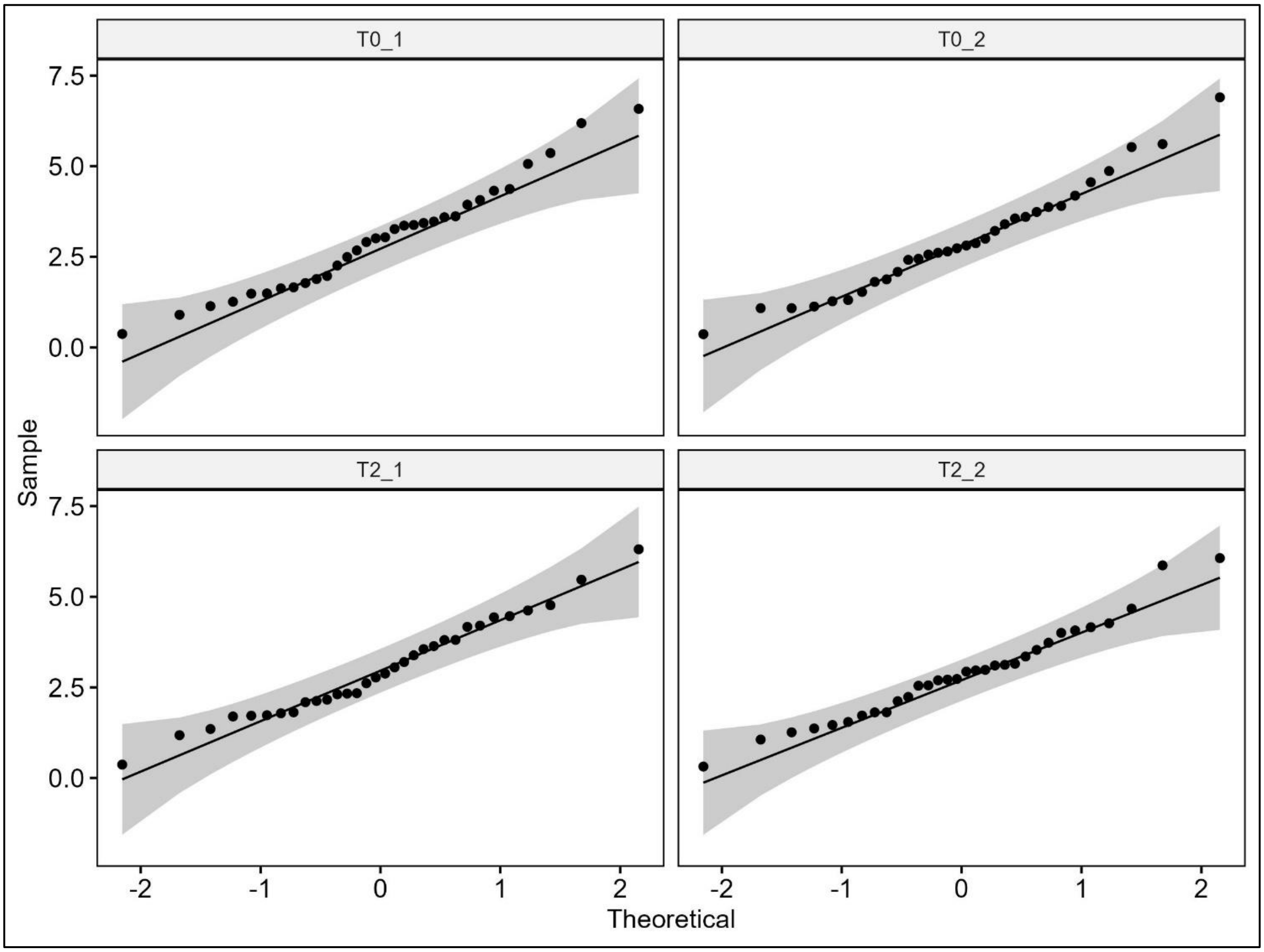

Supplementary Figure 4: Normal distribution of root mean square (RMS) of the right upper trapezius (TrapR) at measurement times. T0 = first competitive session, T2 = second competitive session, _1 = first game, _2 = last game.

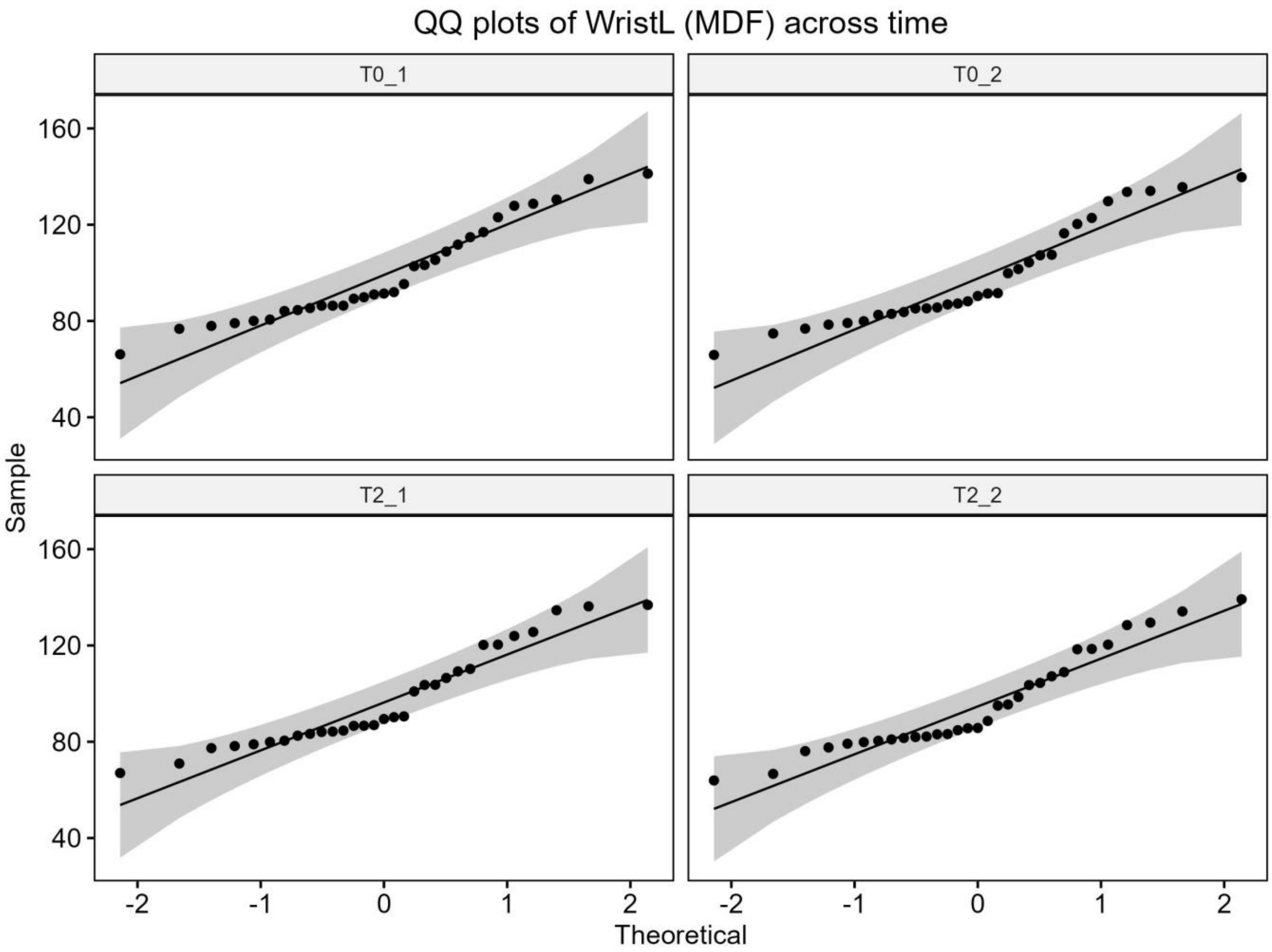

Supplementary Figure 5: Normal distribution of median frequency (MDF) of the left wrist extensor (WristL) at measurement times. T0 = first competitive session, T2 = second competitive session, _1 = first game, _2 = last game.

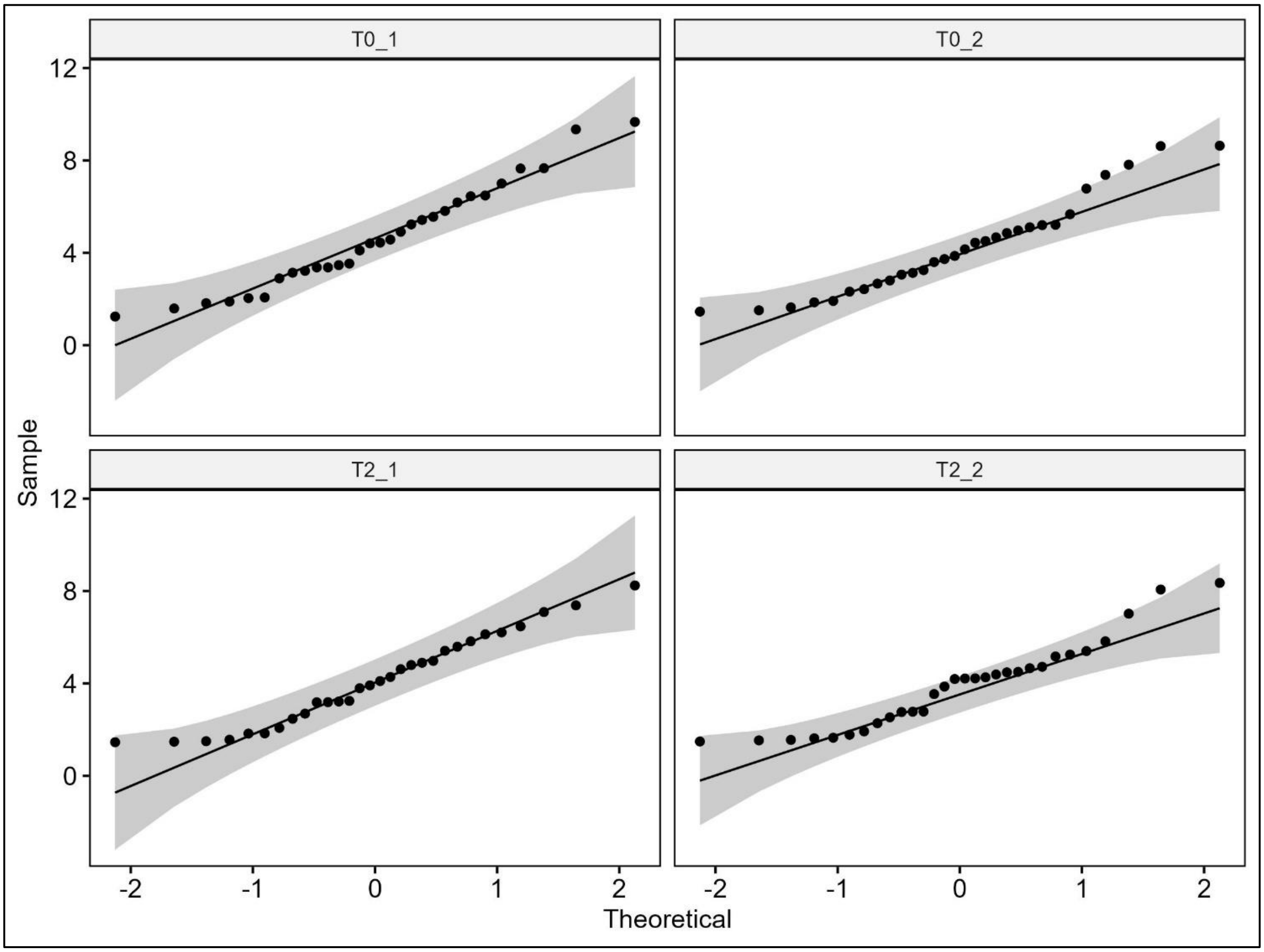

Supplementary Figure 6: Normal distribution of root mean square (RMS) of the left wrist extensor (WristL) at measurement times. T0 = first competitive session, T2 = second competitive session, _1 = first game, _2 = last game.

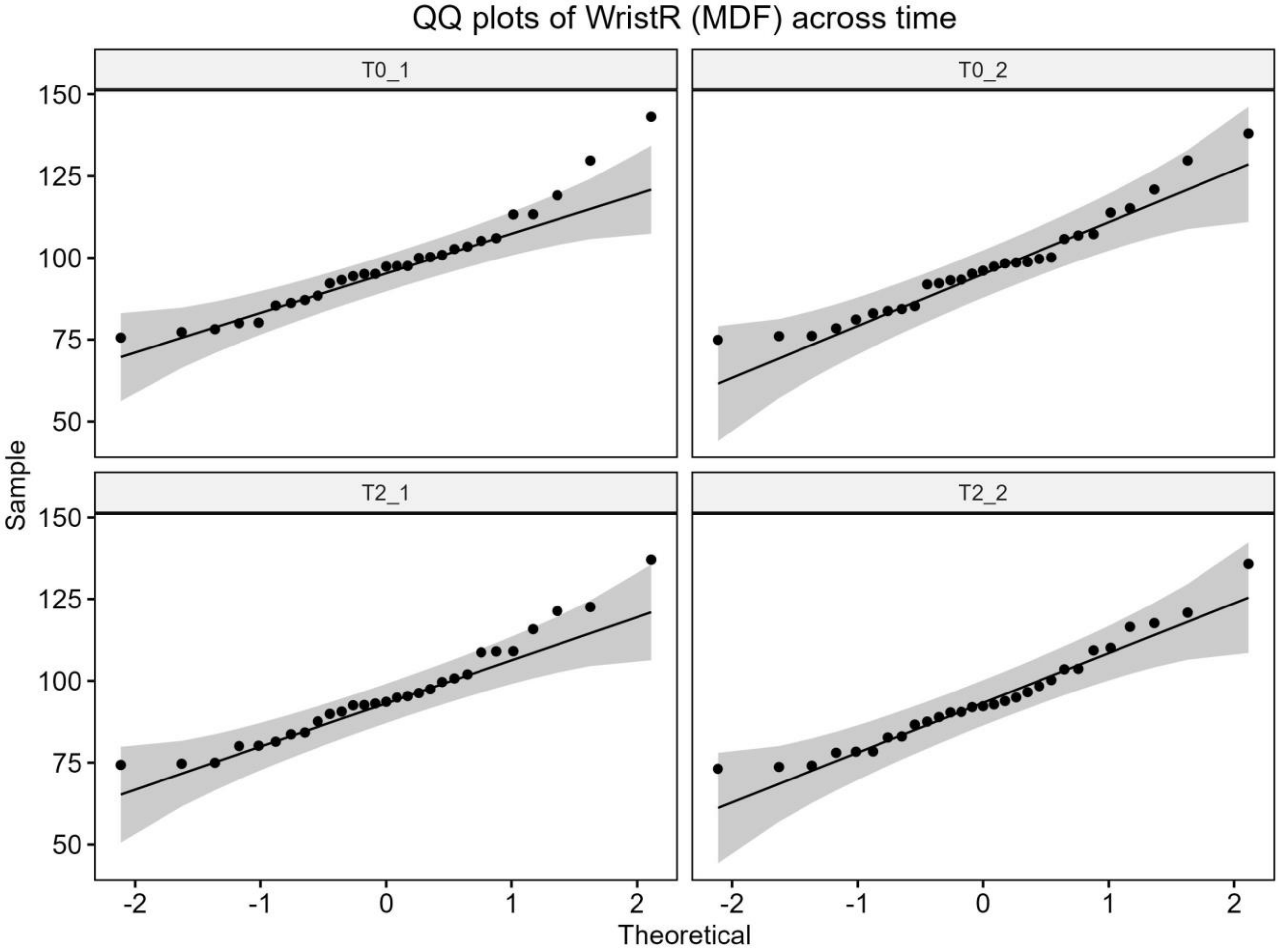

Supplementary Figure 7: Normal distribution of median frequency (MDF) of the right wrist extensor (WristR) at measurement times. T0 = first competitive session, T2 = second competitive session, _1 = first game, _2 = last game.

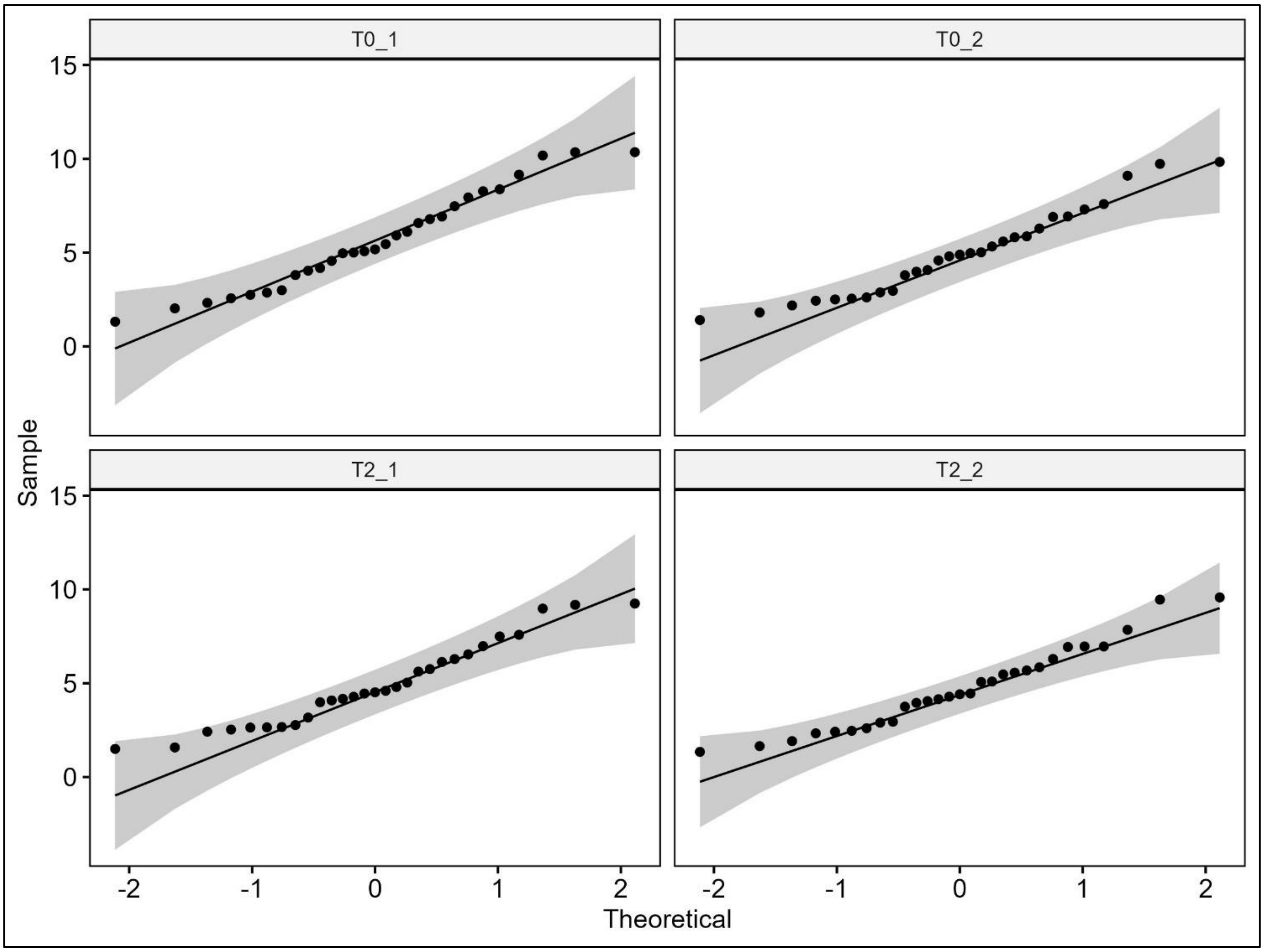

Supplementary Figure 8: Normal distribution of root mean square (RMS) of the right wrist extensor (WristR) at measurement times. T0 = first competitive session, T2 = second competitive session, _1 = first game, _2 = last game.

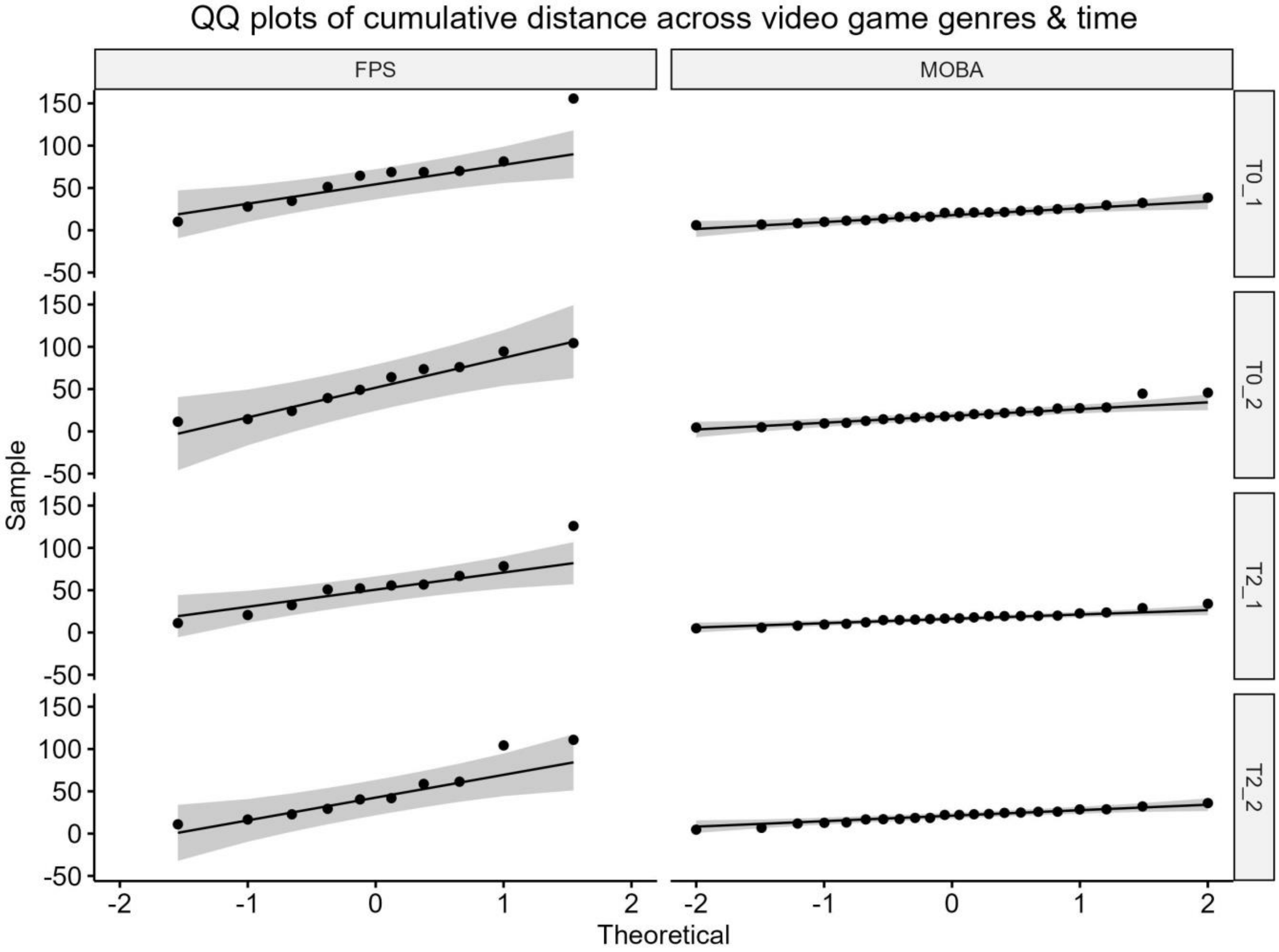

Supplementary Figure 9: Normal distribution of the cumulative distance for video game genres and across measurement times. FPS = First-Person Shooter, MOBA = Multiplayer Online Battle Arena, T0 = first competitive session, T2 = second competitive session, _1 = first game, _2 = last game.

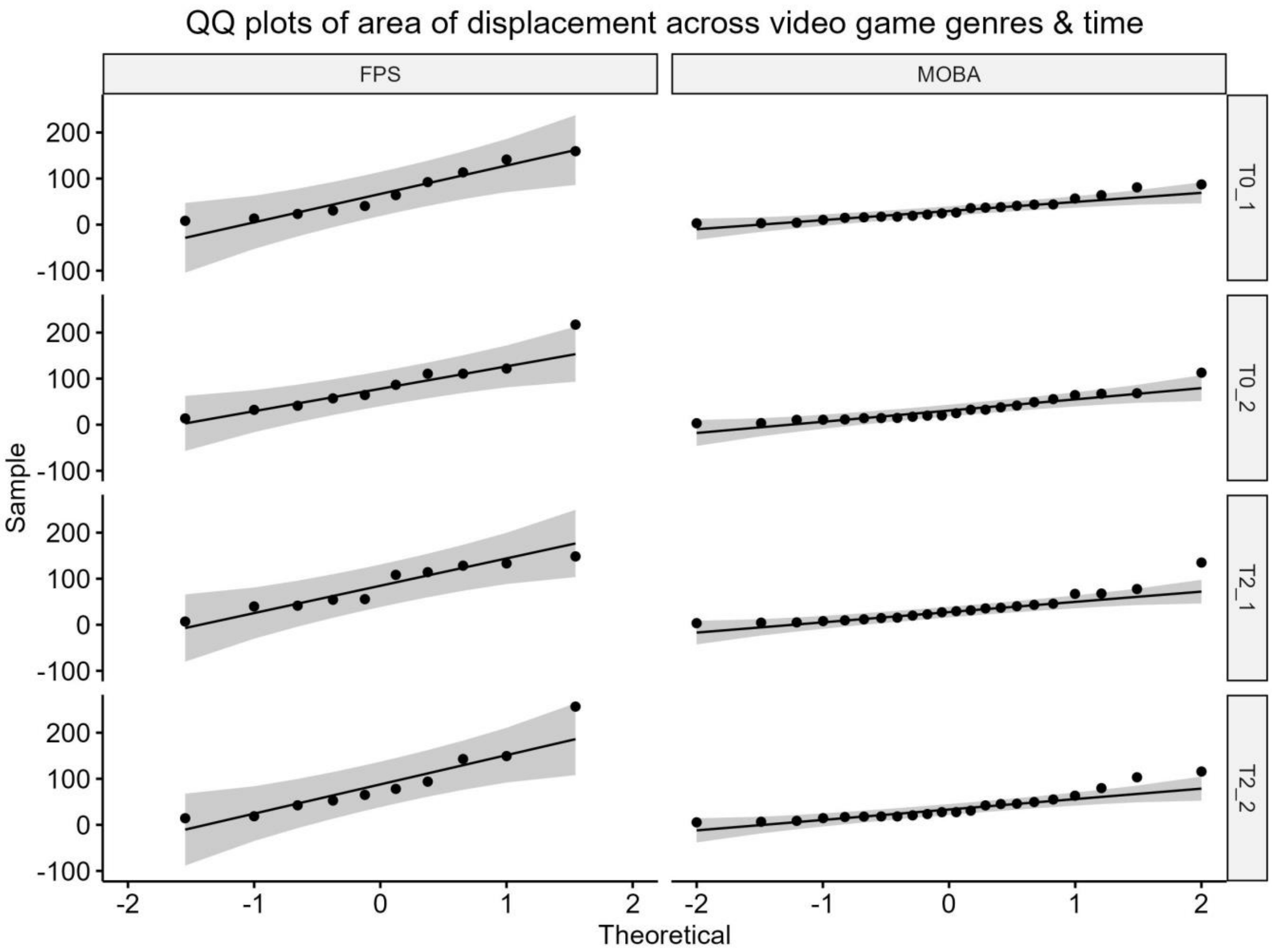

Supplementary Figure 10: Normal distribution of the area of displacement for video game genres and across measurement times. FPS = First-Person Shooter, MOBA = Multiplayer Online Battle Arena, T0 = first competitive session, T2 = second competitive session, _1 = first game, _2 = last game.

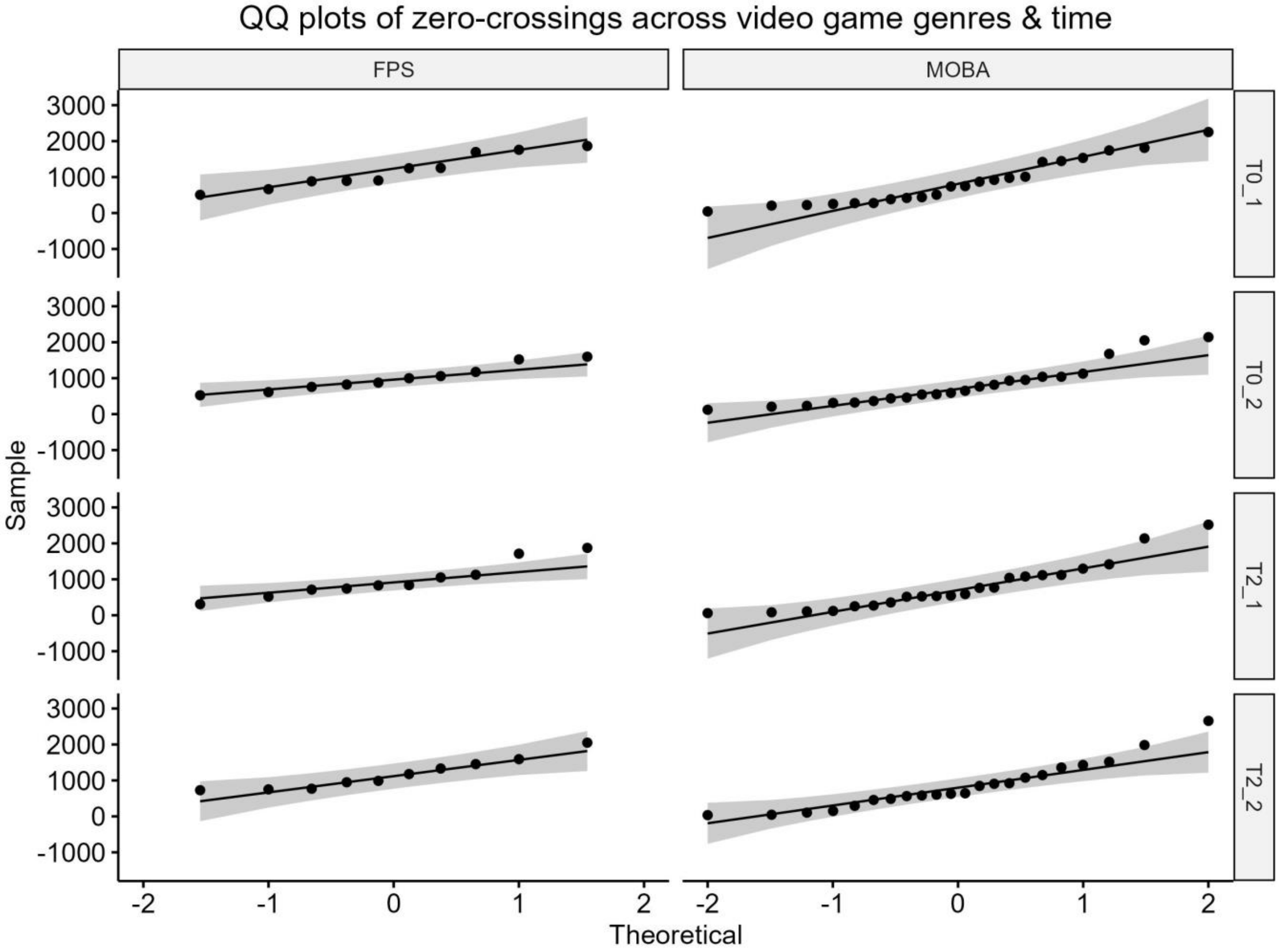

Supplementary Figure 11: Normal distribution of the velocity zero-crossings for video game genres and across measurement times. FPS = First-Person Shooter, MOBA = Multiplayer Online Battle Arena, T0 = first competitive session, T2 = second competitive session, _1 = first game, _2 = last game.

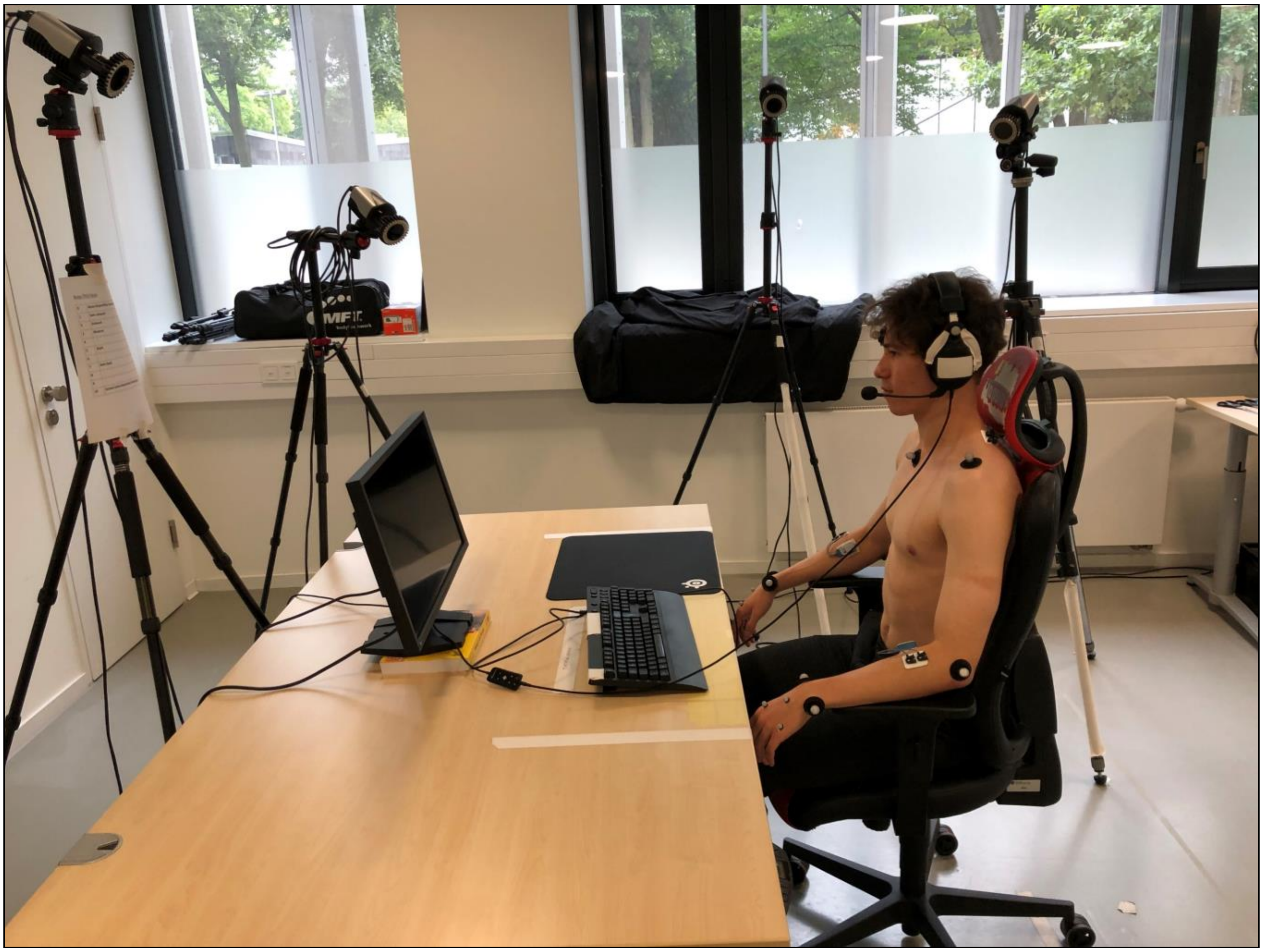

Supplementary Figure 12: Lateral photograph of the experimental setup. Note: The original chair used during the measurements was not available at the time of the photo, a different chair was used for illustrative purposes.

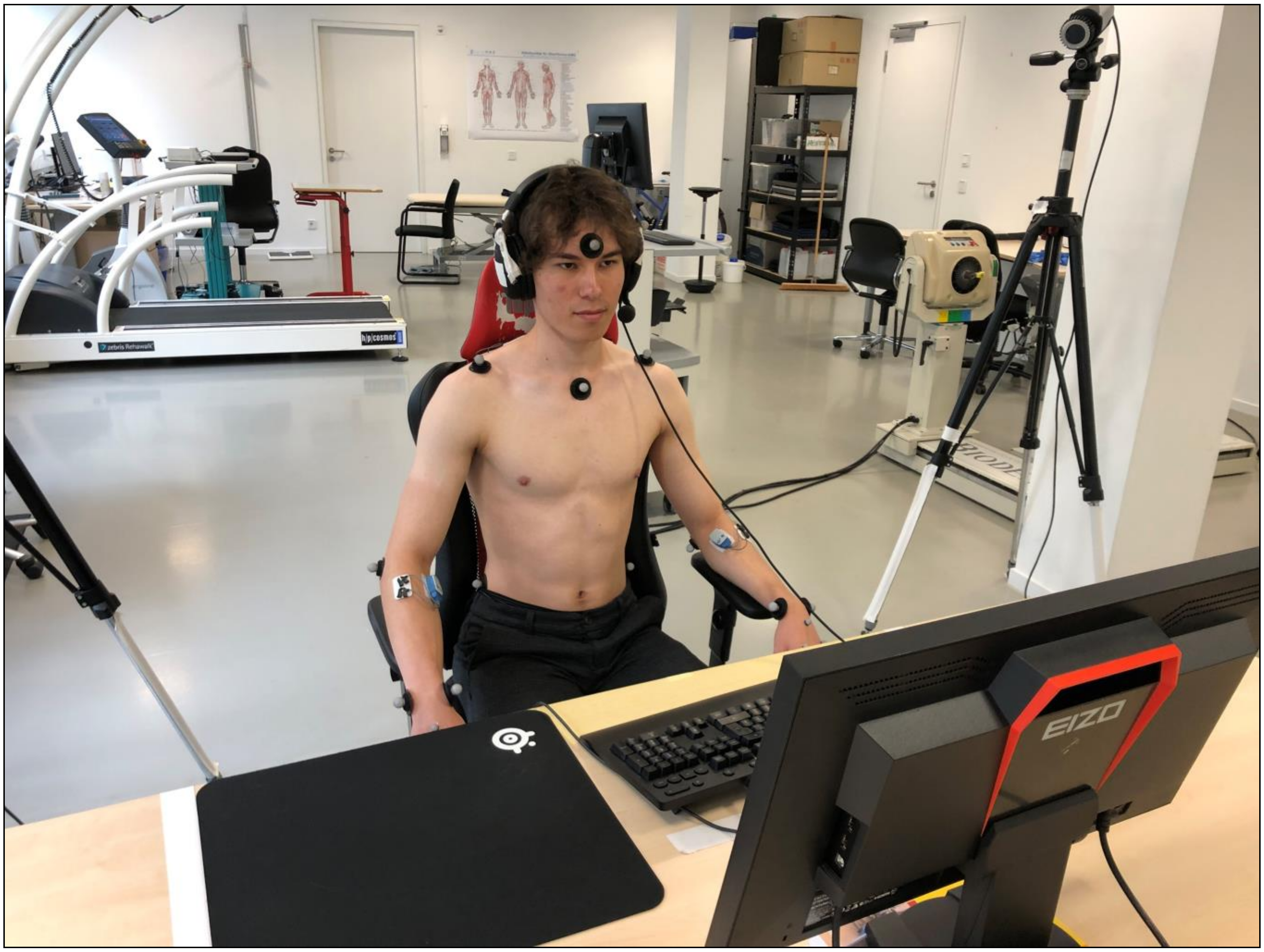

Supplementary Figure 13: Frontal photograph of the experimental setup. Note: The original chair used during the measurements was not available at the time of the photo, a different chair was used for illustrative purposes.

2.2 Supplementary Tables

Supplementary Table 1: Gaming Setup.

**Computer**
• Processor: Intel Core i7 13700KF - 3.4 GHz<br>• Motherboard: ASUS TUF GAMING B660M-<br>• RAM: 2x Crucial DDR4 - 16 GB<br>• Graphic card: ASUS TUF Gaming Radeon RX 6950 XT
**Hardware**
• Monitor: EIZO Foris FG2421<br>• Mouse: Logitech G PRO X SUPERLIGHT<br>• Mouse pad: SteelSeries QcK+<br>• Keyboard: Logitech Prodigy G213<br>• Headset: HyperX Cloud II

Supplementary Table 2: Post-hoc analysis for TrapR (MDF).

time 1	time 2	df	p	p.adjust	effsize	magnitude
T0_1	T0_2	30	0.301	1	0.188879	negligible
T0_1	T2_1	30	0.037	0.223	0.391812	small
T0_1	T2_2	30	0.013	0.076	0.476762	small
T0_2	T2_1	30	0.042	0.25	0.382172	small
T0_2	T2_2	30	0.012	0.071	0.481885	small
T2_1	T2_2	30	0.502	1	0.122091	negligible

T0 = first competitive session, T2 = second competitive session, _1 = first game, _2 = last game.

Supplementary Table 3: Post-hoc analysis for WristL (MDF).

time 1	time 2	df	p	p.adjust	effsize	magnitude
T0_1	T0_2	30	0.225	1	0.222752	small
T0_1	T2_1	30	0.011	0.067	0.485531	small
T0_1	T2_2	30	0.000286	**0.002**	0.737255	moderate
T0_2	T2_1	30	0.085	0.508	0.320304	small
T0_2	T2_2	30	0.002	**0.01**	0.621205	moderate
T2_1	T2_2	30	0.009	0.054	0.501404	moderate

T0 = first competitive session, T2 = second competitive session, _1 = first game, _2 = last game.

Supplementary Table 4: Post-hoc analysis for WristR (MDF).

time 1	time 2	df	p	p.adjust	effsize	magnitude
T0_1	T0_2	28	0.113	0.678	0.304043	small
T0_1	T2_1	28	0.007	**0.045**	0.53578	moderate
T0_1	T2_2	28	0.00046	**0.003**	0.736449	moderate
T0_2	T2_1	28	0.037	0.221	0.407235	small
T0_2	T2_2	28	0.002	**0.011**	0.636576	moderate
T2_1	T2_2	28	0.000332	**0.002**	0.759148	moderate

T0 = first competitive session, T2 = second competitive session, _1 = first game, _2 = last game.

Supplementary Table 5: Post-hoc analysis for WristL (RMS).

time 1	time 2	df	p	p.adjust	effsize	magnitude
T0_1	T0_2	30	0.01	0.062	0.500278	moderate
T0_1	T2_1	30	0.002	**0.011**	0.626484	moderate
T0_1	T2_2	30	0.000344	**0.002**	0.740358	moderate
T0_2	T2_1	30	0.272	1	0.204301	small
T0_2	T2_2	30	0.004	**0.025**	0.567602	moderate
T2_1	T2_2	30	0.041	0.247	0.390283	small

T0 = first competitive session, T2 = second competitive session, _1 = first game, _2 = last game.

Supplementary Table 6: Post-hoc analysis for WristR (RMS).

**time 1**	**time 2**	**df**	**p**	**p.adjust**	**effsize**	**magnitude**
T0_1	T0_2	29	5.86E-08	**3.52E-07**	1.357359	large
T0_1	T2_1	29	1.72E-06	**1.03E-05**	1.118578	large
T0_1	T2_2	29	3.22E-06	**1.93E-05**	1.075325	large
T0_2	T2_1	29	0.298	1	0.197002	negligible
T0_2	T2_2	29	0.019	0.112	0.46368	small
T2_1	T2_2	29	0.044	0.262	0.392302	small

T0 = first competitive session, T2 = second competitive session, _1 = first game, _2 = last game.
